# Viable offspring obtained from *Prm1-*deficient sperm in mice

**DOI:** 10.1038/srep27409

**Published:** 2016-06-02

**Authors:** Naoki Takeda, Kazuya Yoshinaga, Kenryo Furushima, Kazufumi Takamune, Zhenghua Li, Shin-ichi Abe, Shin-ichi Aizawa, Ken-ichi Yamamura

**Affiliations:** 1 Division of Developmental Genetics, Institute of Resource Development and Analysis, Kumamoto University, 2-2-1 Honjo, Kumamoto 860-0811, Japan; 2 Department of Anatomy, Graduate School of Health Sciences, Kumamoto University, 4-24-1 Kuhonji, Kumamoto 862-0975, Japan; 3 Department of Molecular Cell Biology and Molecular Medicine, Institute of Advanced Medicine, Wakayama Medical University, 811-1 Kimiidera, Wakayama 641-8509, Japan; 4 Department of Biological Sciences, Graduate School of Science and Technology, Kumamoto University, 2-39-1 Kurokami, Kumamoto 860-8555, Japan; 5 Department of Histology and Embryology, Harbin Medical University, Harbin 150081, China; 6 Kumamoto Health Science University, 325 Izumi-machi, Kita-ku, Kumamoto 861-5598, Japan; 7 Center for Developmental Biology, RIKEN Kobe, 2-2-3 Minatojima Minamimachi, Chuo-ku, Kobe 650-0047, Japan; 8 Yamamura Project Laboratory, Institute of Resource Development and Analysis, Kumamoto University, Kumamoto 860-0811, Japan

## Abstract

Protamines are expressed in the spermatid nucleus and allow denser packaging of DNA compared with histones. Disruption of the coding sequence of one allele of either *protamine 1* (*Prm1*) or *Prm2* results in failure to produce offspring, although sperm with disrupted *Prm1* or *Prm2* alleles are produced. Here, we produced *Prm1-*deficient female chimeric mice carrying *Prm1-*deficient oocytes. These mice successfully produced *Prm1*^+/−^ male mice. Healthy *Prm1*^+/−^ offspring were then produced by transferring blastocysts obtained via *in vitro* fertilization using zona-free oocytes and sperm from *Prm1*^+/−^ mice. This result suggests that sperm lacking *Prm1* can generate offspring despite being abnormally shaped and having destabilised DNA, decondensed chromatin and a reduction in mitochondrial membrane potential. Nevertheless, these mice showed little derangement of expression profiles.

Chromosomal DNA is compacted into the microscopic space of the cell nucleus primarily by histones in somatic cells and by protamines in sperm. Histones are highly alkaline proteins, whereas protamines are small arginine-rich proteins. During spermatid differentiation (spermiogenesis), nuclear remodelling and condensation are associated with the sequential displacement of histones by transition proteins and then by protamines, namely protamine 1 (PRM1) and protamine 2 (PRM2)^1^. Whereas PRM1 is present in all mammals and many other species, PRM2 is found in all primates—but only in certain rodents and a few other mammals. Protamines allow for denser packaging of DNA in the spermatozoon compared with histones. Therefore, protamines are believed to play important roles in chromatin condensation, suppression of transcription, protection of the haploid male genome, determination of sperm shape, and production of offspring. Humans and mice have three protamine genes: *Prm1, Prm2*, and *Prm3*. In mice, PRM1 is an arginine- and cysteine-rich protein of 50 amino acids, whereas PRM2 is rich in histidine and has 106 amino acids. It is likely that the *Prm2* gene is derived from a duplication of the *Prm1* gene. Approximately 70% of the protamine in mouse sperm and 50% of that in human sperm is PRM2^2^. *Prm2* has several features that distinguish it from *Prm1*. *Prm2* encodes a precursor protein that binds to DNA and then undergoes proteolytic processing, which results in the removal of approximately 40% of the amino terminus of the molecule to yield a mature form of 63 amino acids^3^. PRM2 also differs from PRM1 in its ability to bind zinc^4^. The relationships between *Prm1* and *Prm2* is not well understood. Gene-knockout experiments have shown that the presence of both *Prm1* and *Prm2* is required for proper spermatid maturation and male fertility in mice^5^. In addition, maintaining the correct proportion of the two protamines in mice is critical for maintaining the integrity of sperm chromatin. Mouse sperm deficient in *Prm2* have increased DNA damage, incomplete chromatin condensation, and other defects that block embryonic development beyond the blastocyst stage^6^. Alterations in the composition and structural organisation of sperm chromatin (to which PRM1 and PRM2 contribute) may affect both fertilisation and early events in embryonic development^7^. *Prm3* encodes a putative 104-amino acid polypeptide with high glutamic acid content^8^ that is localized in the cytoplasm instead of the nucleus^9^. Although the sperm from *Prm3*^−/−^ mice exhibit reduced motility^9^, Martin-Coello *et al.*^10^ found that PRM3 is not a true protamine and is not involved in chromatin condensation.

In humans, an initial mutational analysis of the protamine genes suggested that the presence of pathogenic mutations in these genes was a rare cause of infertility^11^,^12^,^13^,^14^,^15^,^16^,^17^,^18^. However, a single-nucleotide polymorphism (SNP; G197T) that results in an arginine-to-serine change in the highly conserved arginine clusters needed for normal DNA binding has been found in 10% of unrelated infertile patients whose sperm were phenotypically similar to those from protamine-deficient mice^19^.

In mice, Cho *et al.*^5^,^6^ reported that male chimeras produced by an injection of *Prm1*^+/−^ embryonic stem (ES) cells or *Prm2*^+/−^ ES cells into C57BL/6N blastocysts could not transmit either the mutant or wild-type allele derived from the *Prm1*^+/−^ or *Prm2*^+/−^ ES cells to the next generation. These authors also performed intra-cytoplasmic sperm injection using *Prm2*-deficient sperm; however few eggs were able to develop to the blastocyst stage. Cho *et al.*^5^,^6^ were unable to obtain *Prm1*^+/−^ or *Prm2*^+/−^ heterozygous mice using natural mating or intracytoplasmic sperm injection.

Here, for the first time to our knowledge, we successfully obtained female chimeric mice carrying *Prm1-*deficient oocytes, and subsequently obtained *Prm1*^+/−^ male mice. Healthy *Prm1*^+/−^ offspring were born by transferring blastocysts obtained by *in vitro* fertilization using zona-free oocytes with sperm from these *Prm1*^+/−^ mice. This achievement suggests that the DNA is not severely damaged in sperm lacking *Prm1*. Although the sperm are abnormally shaped and exhibit DNA destabilisation, chromatin decondensation, and reduced mitochondrial membrane potential, there is little derangement of expression profiles.

## Results

### Generation of *Prm1* heterozygous mice

Following electroporation with the targeting vectors, 569 neo-resistant clones were screened for targeted recombination using Southern blot analysis. With probe A, three clones (Nos. A36, F6, and ϕ18) yielded 10.9-kb and 9.6-kb bands when digested with *Eco*RI/*Eco*RV (Supplementary Fig. 1). All three clones yielded 9.0-kb and 7.7-kb bands or 7.3-kb and 6.0-kb bands when digested with *ApaL1* and *Sca*I, respectively (Supplementary Fig. 1). These patterns indicated the presence of the targeted allele.

As previously reported^5^, heterozygous *Prm1-*deficient mice (*Prm1*^+/−^) cannot be obtained by natural mating between chimeric male mice produced using targeted ES cells and wild-type mice. Because we expected that oocyte formation might not be affected by protamine deficiency, we used TT2-XO ES cells^20^ to determine whether we could generate female *Prm1*^+/−^ mice. Three independent targeted ES clones were injected into eight-cell stage embryos to generate chimeric mice. Female chimeric mice from three clones successfully produced male and female *Prm1*^+/−^ offspring. Female F1 mice from ES clone No. A36 were backcrossed to C57BL/6J for a further six generations. All male *Prm1*^+/−^ mice used in the following experiments were from the sixth generations or later.

As expected, *Prm1*^+/−^ males did not sire offspring by natural mating. We therefore attempted *in vitro* fertilization (IVF) using sperm from *Prm1*^+/−^ mice. However, no fertilised eggs were obtained. Because sperm from the *Prm1*^+/−^ mice might not penetrate the zona pellucida, we performed IVF with zona-free oocytes. This procedure yielded fertilised eggs (fertilisation rate 73%; 62/85). Eighty fertilised eggs were then cultured *in vitro*. Of these, 34 developed to the two-cell stage and 23 to the blastocyst stage. These 23 blastocysts were transferred into uteri and yielded 16 foetuses on embryonic day 12.5. Surprisingly, six embryos (37.5%) were identified as *Prm1*^+/−^ mice by polymerase chain reaction (PCR). We then further investigated whether the *Prm1*^+/−^ males could sire offspring. In an initial experiment, we transferred two-cell-stage embryos into the oviducts of foster mothers at 0.5-days post-coitus. However, no offspring were obtained. Next, we transferred 50 four-cell-stage embryos into the oviducts of foster mothers at 0.5 days post-coitus. Seven mice were born, three of which were *Prm1*^+/−^. These results clearly suggest that *Prm1-*deficient sperm can generate offspring. Subsequently, *Prm1*^+/−^ offspring were obtained either by transferring blastocysts developed from *in vitro* fertilised eggs into the uterus of foster mothers at 2.5 days post-coitus or by mating female *Prm1*^+/−^ mice with male *Prm1*^+/+^ mice.

*Prm1*^+/+^ males (n = 6) and *Prm1*^+/−^ males (n = 7) did not differ in terms of external appearance, body weight, testis weight, or epididymis weight (Supplementary Table 1).

### Basic nuclear protein expression

**Figure 1:**
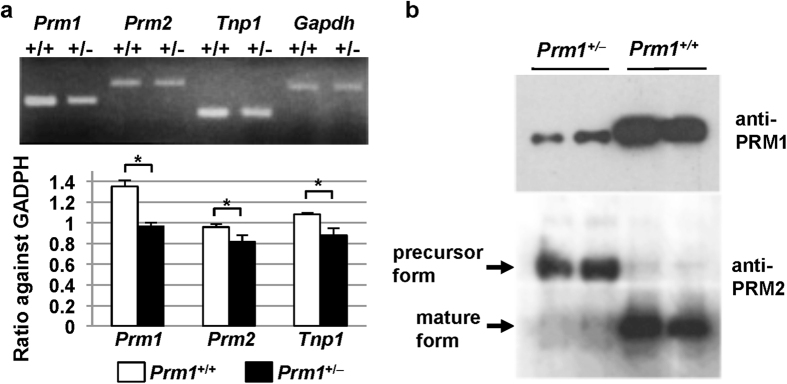
Targeted mutation at the *Prm1* locus. (**a**) RT-PCR analysis of the mRNAs encoding basic proteins in the cauda epididymis and vas deferens. (**b**) Western blot analysis. The levels of PRM1 were reduced and the levels of the precursor forms of PRM2 were elevated in the *Prm1*^+/−^ *mice*. Statistical analyses were performed using unpaired Student’s t-tests (*P < 0.05, **P < 0.01).

We performed the following analyses on the *Prm1*^+/−^ mice. To assess the effect of *Prm1* deficiency, the expression levels of the sperm-specific nuclear protein genes *Prm1*, *Prm2*, and *Tnp1* in sperm from the cauda epididymis and vas deferens were analysed using RT-PCR. The expression levels of all of these genes were slightly reduced (Fig. 1a). Western blot analyses were performed using either anti-PRM1 or anti-PRM2 antibodies and showed that the level of PRM1 in *Prm1*^+/−^ mice was reduced to approximately half that in *Prm1*^−/−^ mice (Fig. 1b). As expected, the precursor form of PRM2 was detected in the *Prm1*^+/−^ mice, whereas production of the mature form of RPM2 was greatly reduced (Fig. 1b).

### Protamine status in *Prm1* ^+/−^ mice

**Figure 2:**
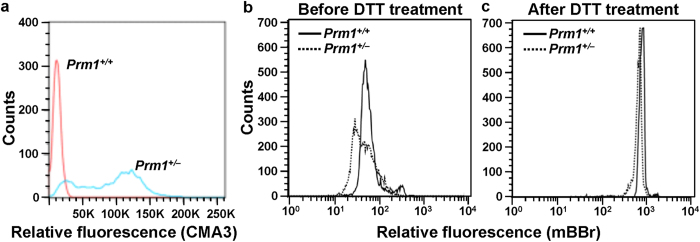
Protamine deficiency. (**a**) CMA3 assay. Flow cytometric plots showing nuclei of spermatozoa stained with CMA3. CMA3 is a fluorochrome that detects protamine deficiency in loosely packed chromatin. (**b,c**) Thiol labelling assay. Mean fluorescence intensities in the *Prm1*^+/−^ sperm before and after DTT treatment were lower than those in the *Prm1*^+/+^ sperm, indicating a lower content of free and total thiols in the *Prm1*^+/−^*sperm*.

Chromomycin A3 (CMA3) competes with protamines for binding to the minor groove of DNA^21^. Therefore, CMA3 is a simple and useful tool for assessing the packaging of sperm chromatin and allows indirect visualization of protamine deficiency. Flow cytometry histogram analysis showed low fluorescence intensity with a single peak in *Prm1*^+/+^ mice, indicating normal chromatin packaging. In *Prm1*^+/*−*^ mice, biphasic peaks, with one at the same fluorescence and the other at a stronger fluorescence compared with the wild-type samples, were observed, indicating the presence of two types of sperm in *Prm1*^+/−^ mice: one with the same PRM content as the wild-type sperm and the other with a lower PRM content (Fig. 2a).

**Figure 3:**
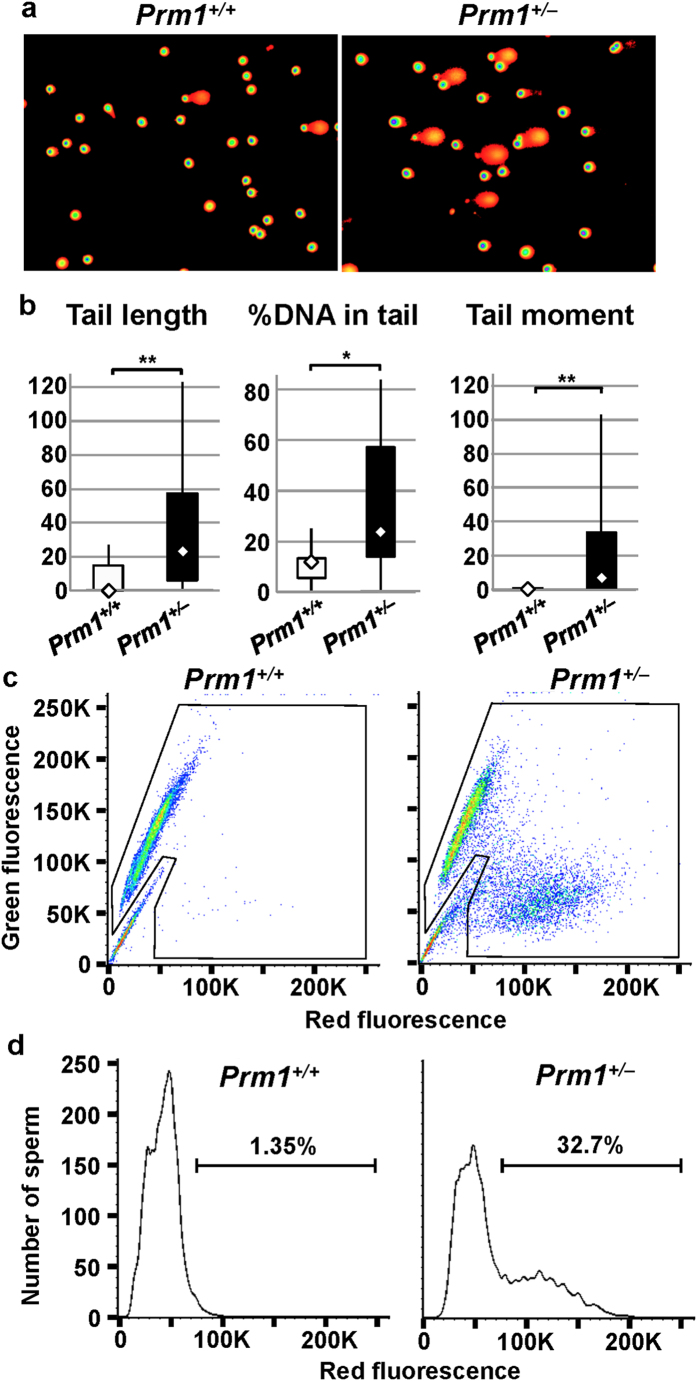
DNA destabilization. (**a,b**) Comet assay. a. Photographs of epididymal sperm comets. (**b**) DNA in the *Prm1*^+/−^ sperm was destabilised, as judged by the increased indices of tail length, the %DNA in the tail, and the tail moment. (**c**) SCSA cytogram. SCSA using acridine orange staining showed high levels of green fluorescence but little red fluorescence in the *Prm1*^+/+^ sperm and low levels of green fluorescence and high levels of red, fluorescence in the *Prm1*^+/−^ sperm, suggesting a reduction of sperm DNA integrity in the *Prm1*^+/−^ *mice*. (**d**) Histograms of the numbers of sperm showing red fluorescence. Sperm of *Prm1*^+/−^ mice showed biphasic peaks. Percentages of cells outside the main population are also shown. Comet parameters were analysed using the Mann-Whitney U-Test (*P < 0.05, **P < 0.01).

The thiol-disulphide status of spermatozoa can be measured using the fluorescent thiol-labelling agent monobromobimane (mBBr). Because protamine is a thiol protein and thiol oxidation occurs in protamine during sperm epididymal maturation, mBBr can be used to determine the sperm protamine content and thiol-disulphide status in *Prm1*^+/−^ mice. mBBr-labelled spermatozoa from the cauda epididymis and vas deferens were analysed via flow cytometry before and after 1,4-dithiothreitol (DTT) treatment. The mean mBBr fluorescence intensity in the *Prm1*^+/+^ mice was significantly higher than that in the *Prm1*^+/−^ mice before DTT treatment (*P* < 0.05; Fig. 2b), indicating that the *Prm1*^+/−^ mice had a lower content of free thiols. Interestingly, the flow cytometry histogram of the *Prm1*^+/−^ mice was broad or biphasic, whereas that of the *Prm1*^+/+^ mice had a single peak, indicating that the *Prm1*^+/−^ mice had two types of sperm: one with low free thiol and the other with the same thiol level as the wild-type sperm. The total amount of sperm thiols reflects the total amount of PRM and can be measured after treatment with DTT. The total thiol intensity of the *Prm1*^+/−^ mice was approximately 80% of that of the *Prm1*^+/+^ mice after the DTT treatment (*P* < 0.01; Fig. 2c). These results suggest that *Prm1*^+/−^ mice have sperm populations with two different disulphide states (arising from the absence or presence of *Prm1*), thereby resulting in low levels of free and total thiols. Alternatively, the biphasic peaks might be a secondary effect of altered nuclear integrity or degenerated PRM1-deficient sperm because sperm in the cauda epididymis of *Prm1*^+/−^ mice are often phenotypically abnormal and their numbers are reduced. In fact, the heterogeneous appearance on comet assay of sperm from *Prm1*^+/−^ mice (Fig. 3a) is consistent with this explanation.

### DNA damage and stability in sperm from *Prm1* ^+/−^ mice

Sperm recovered from the cauda epididymis and vas deferens were analysed for DNA damage using a comet assay. Sperm from the *Prm1*^+/−^ mice showed larger comets than sperm from the wild-type mice (Fig. 3a). The comet assay parameters (median and interquartile range) for the *Prm1*^+/+^ mice (*n* = 12) were as follows: tail length, 0 (0 to 15); % tail DNA, 11.9 (5.60 to 13.3); and tail moment, 0 (0 to 1.1). By contrast, the parameters for the *Prm1*^+/−^ mice (*n* = 19) were as follows: tail length, 23.0 (6.0 to 57.5); % tail DNA, 22.8 (14.0 to 57.2); and tail moment, 7.4 (0.8 to 33.4) (Fig. 3b). There were significant differences between the two genotypes in terms of tail length and tail moment (*P* < 0.01) as well as the % tail DNA (*P* < 0.05).

A sperm chromatin structure assay (SCSA) was used to examine the sperm DNA integrity via flow cytometry of acridine orange (AO)–stained sperm. To assess the susceptibility of sperm chromatin to low pH–induced denaturation, the SCSA was performed by a previously described method^22^. AO intercalates with intact double-stranded DNA to emit green fluorescence, whereas the dye emits red fluorescence when associated with RNA or denatured DNA. Examination of the fluorescence spectra of sperm from the *Prm1*^+/+^ mice showed one group that emitted mostly green fluorescence, whereas the fluorescence spectra of the sperm of the *Prm*^+/−^ mice showed two groups—one emitting green and the other red fluorescence (Fig. 3c)—suggesting the presence of two types of sperm. Sperm from the *Prm1*^+/−^ mice showed biphasic peaks of red fluorescence, which represent large numbers of sperm with denatured DNA (Fig. 3d). The percentage of cells outside the main population was 1.35% in the *Prm1*^+/+^ mice and 32.7% in the *Prm1*^+/−^ mice (Fig. 3d). The results of both the comet assay and the SCSA show reduced chromatin integrity, which destabilises the DNA packaging of the sperm of *Prm1*^+/−^ mice.

### Sperm morphology

**Figure 4:**
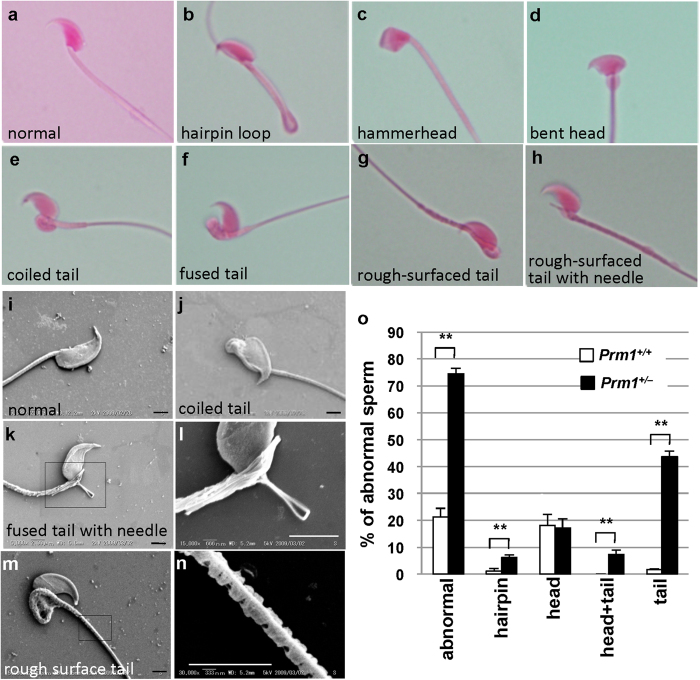
Sperm morphology, as indicated by eosin staining and scanning electron microscopy. (**a**–**h**) Eosin staining. (**i**–**n**) SEM. l, higher magnification of (**k**). (**n**), Higher magnification of (**m,o**), frequencies of abnormal spermatozoa. Scale bar: 2 μm. Statistical analyses were performed using unpaired Student’s t-tests (*P < 0.05, **P < 0.01).

To examine the sperm morphology, we first performed eosin staining. Abnormalities can be classified depending on the site of abnormality (i.e., head or tail). A typical sperm is shown in Fig. 4a. Hairpin loops (Fig. 4b) were frequently observed in the sperm of both *Prm1*^+/+^ and *Prm1*^+/−^ mice. Head abnormalities included a “hammer head” appearance (Fig. 4c) and a tail attached to a bent head (Fig. 4d). Abnormalities were more frequent in the tail region of *Prm1*^+/−^ mice and included coiled (Fig. 4e), fused (Fig. 4f), and rough-surfaced (Fig. 4g,h) tails. Tail abnormalities were often accompanied by a needle-like structure (Fig. 4h). Bent heads were often associated with head distortion.

Similar sperm abnormalities were observed via scanning electron microscopy (SEM) (Fig. 4i [normal sperm] to l); they included coiled tail (Fig. 4j) and a fused tail with a needle-like structure (Fig. 4k,l). SEM images of a rough-surfaced tail are shown in Fig. 4m,n. We observed fine fibres protruding from the periphery of the broken tails (Fig. 4k,l).

Eosin staining revealed that 22.3% of the epididymal spermatozoa from the *Prm1*^+/+^ mice were abnormal, compared with 74.6% of those from the *Prm1*^+/−^ mice (*P* < 0.01; Fig. 4o). In addition, the hairpin, head + tail and tail sperm abnormalities were all significantly more common in the *Prm1*^+/−^ mice than in the *Prm1*^+/+^ mice (*P* < 0.01; Fig. 4o). Therefore, the haploid insufficiency of PRM1 increased the rate of abnormal sperm morphologies.

**Figure 5:**
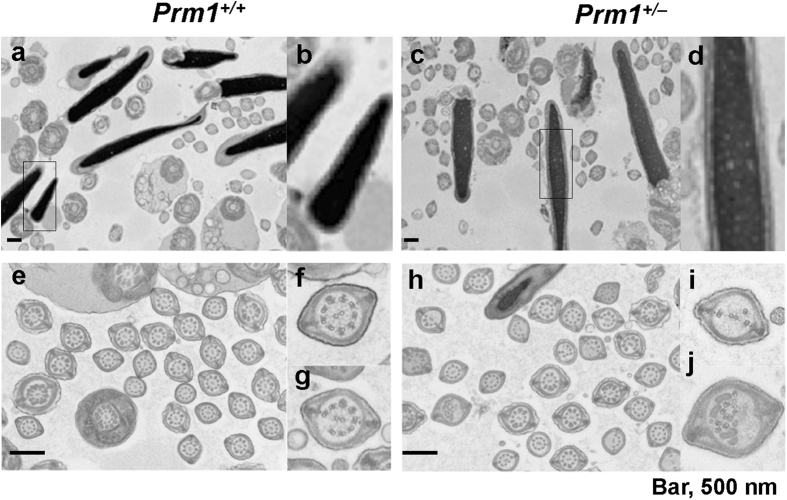
DNA decondensation and flagellum abnormalities under TEM. (**a**) *Prm1*^+/+^ sperm heads showing normal electron density. (**b**) Magnified view of the sperm head in (**a**). (**c**) *Prm1*^+/−^ sperm head showing heterogeneous electron density. (**d**) Magnified view of the sperm head in (**c**). (**e**) Ultrastructure of the *Prm1*^+/+^ sperm flagellum showing a normal microtubule structure. (**f,g**) Magnified views of (**e**). (**h**) Ultrastructure of the *Prm1*^+/−^ sperm flagellum, showing an abnormal structure (arrows). (**i,j**) Magnified figure of the sperm head in (**h**). Scale bar: 500 nm.

Transmission electron microscopy (TEM) was used to determine the appearance of chromatin, the organisation and placement of flagellar components, and the association between the acrosome and the nucleus. The sperm chromatin was tightly compacted in the wild-type sperm as shown by the density of staining observed on electron microscopy (Fig. 5a,b), although it was heterogeneous with clear spots in sperm from *Prm1*^+/−^ mice (Fig. 5c,d), as previously reported^6^,^23^. The flagellum of normal sperm is a bundle of nine fused pairs of microtubule doublets surrounding two central single microtubules—the so-called “9+2” structure (Fig. 5e,f). Almost half of the microtubules in the *Prm*^+/−^ sperm showed a disorganised arrangement of microtubule doublets or a loss of multiple doublets (Fig. 5g–j).

### Characterisation of sperm

**Figure 6:**
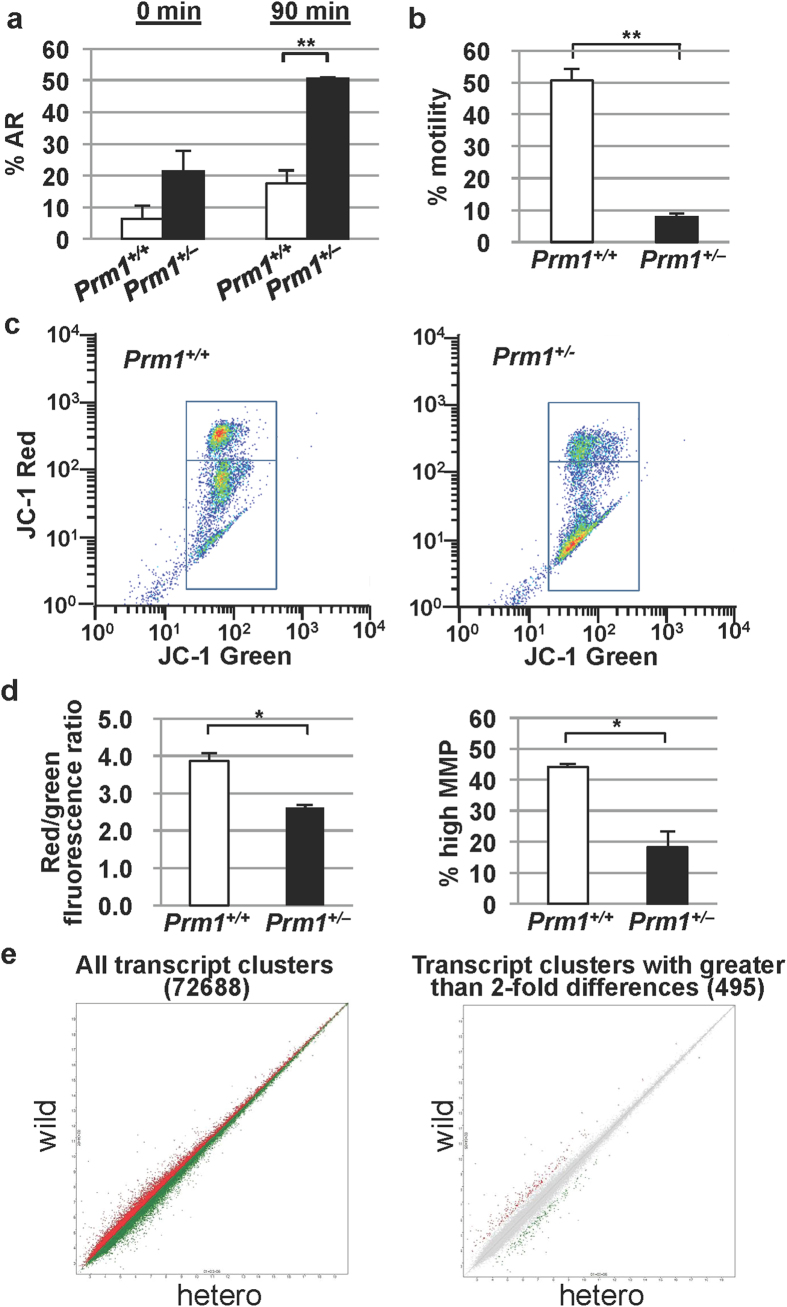
Characterisation of epididymal sperm. (**a**) Acrosome reaction (AR). The percentage of sperm with spontaneous ARs in the *Prm1*^+/−^ mice was significantly increased after sperm incubation for 90 min. (**b**) Motility of spermatozoa, showing a significant reduction of motility in the *Prm1*^+/−^ mice. (**c**) Cytogram of the mitochondrial inner transmembrane potential. JC-1 labels mitochondria with high membrane potential orange (JC-1 aggregates) and mitochondria with low membrane potential green (JC-1 monomers). Orange-stained sperm appear in the upper right quadrant; green-stained spermatozoa appear in the lower right quadrant. (**d**) Histogram of JC-1 staining. The red/green fluorescence ratio in the *Prm1*^+/−^ mice was significantly lower than that in the *Prm1*^+/+^ mice. The percentage of sperm with a high mitochondrial membrane potential (MMP) in the *Prm1*^+/−^ mice was significantly lower than that in the *Prm1*^+/+^ mice. (**e**) DNA microarray analysis. Both the scatter plot of the entire transcript cluster (72,688) and the scatter plot of the transcripts clusters with more than a 200% difference (495) between the *Prm1*^+/+^ and *Prm1*^+/−^ mice showed similar expression profiles in the two mouse genotypes. Statistical analyses were performed using unpaired Student’s t-tests (*P < 0.05, **P < 0.01).

The acrosome reaction (AR) is generally believed to be necessary for penetration of the zona pellucida. Because sperm from *Prm1*^+/−^ mice cannot fertilise eggs with a zona pellucida, we examined the AR status in sperm from the *Prm1*^+/−^ mice. Coomassie brilliant blue G250 stain was used to directly visualise the acrosome under a bright field microscope. At the start of incubation (0 min), there was a tendency towards spontaneous AR, although the percentage of sperm with AR did not differ significantly and presented values of 6.3% ± 4.3% in the *Prm1*^+/+^ mice and 21.5% ± 6.4% in *Prm1*^+/−^ mice (Fig. 6a). Following sperm incubation for 90 min in the presence of Ca^2+^ ionophores, the percentage of sperm with AR in the *Prm1*^+/−^ mice was 50.6% ± 0.4%, which was significantly higher than that in the *Prm1*^+/+^ mice (17.4% ± 4.3%; *P* < 0.01, Fig. 6a), suggesting that the AR was highly induced in the *Prm1*^+/−^ sperm.

We did not use computer-assisted semen analysis to examine the motility of spermatozoa from the *Prm1*^+/−^ mice because it was too weak to detect by this method. Instead, we used phase-contrast microscopy. The motility of the spermatozoa from the *Prm1*^+/+^ mice was 50.7% and that of the spermatozoa from the *Prm1*^+/−^ mice was 7.8% (*P* < 0.01, Fig. 6b). The motility of sperm from the *Prm1*^+/−^ mice was thus greatly reduced, and there was substantial variation in the velocity of both the sperm head along a straight line ans the flagellar beating (data not shown).

A reduction in the mitochondrial membrane potential (MMP) of sperm is reportedly associated with a reduction in sperm motility^24^. The MMP can be measured using 5,5′,6,6′-tetrachloro-1,1′,3,3′-tetraethylbenzimidazolcarbocyanine iodide (JC-1) fluorescence. JC-1 labels mitochondria with a high membrane potential red and mitochondria with a low membrane potential green. Red-stained sperm appeared in the upper region of our plots and were considered healthy, whereas green-stained spermatozoa appeared in the lower region and were considered unhealthy or dead (Fig. 6c). The mean red-to-green fluorescence ratio (3.86 ± 0.22) in the wild-type sperm was significantly higher than that in the *Prm1*^+/−^ mice (2.61 ± 0.06; *P* < 0.05, Fig. 6d). The mean percentage of the highly fluorescent populations (44.1% ± 1.0%) in the *Prm1*^+/+^ sperm was significantly higher than that in the *Prm1*^+/−^ sperm (18.2% ± 5.2%; *P* < 0.05, Fig. 6d), indicating that the percentage of unhealthy or dead sperm in the *Prm1*^+/−^ mice was higher than that in the *Prm1*^+/+^ mice.

Following dataset filtration, dataset condensation via replacement of replicate spots with their averages, and control spot removal, the expression dataset contained 72,688 probes. As expected, the expression levels of *Prm1*, *Prm2*, *Tnp1*, and *Tnp2* in the *Prm1*^+/−^ epididymis were lower (72.46%, 67.11%, 75.76%, and 65.79%, respectively) than those in the *Prm1*^+/+^ epididymis. This finding is consistent with the data on the expression levels of sperm-specific nuclear protein genes (Fig. 1), thus confirming the reliability of the quality of the epididymal mRNA. The expression profiles in the *Prm1*^+/−^ epididymis differed by more than 200% from those in the *Prm1*^+/+^ epididymis in only 495 (0.68%) of the 72,688 probe sets, suggesting that the expression profiles in the two genotypes were similar (Fig. 6e) and that there was no obvious derangement of expression in the absence of PRM1.

## Discussion

We demonstrated here that *Prm1*^+/−^ sperm could fertilise zona-free oocytes *in vitro*, and *Prm1*^+/−^ mice born from these embryos did not exhibit phenotypic abnormalities except sperm morphological abnormalities. As expected, *Prm1*^+/−^ sperm showed chromatin decondensation and destabilisation, abnormal morphology, reduced motility, and enhanced AR; however, there was little derangement of gene-expression profiles in the sperm.

Previous studies^5^,^6^ have demonstrated that *Prm1* or *Prm2* male or female chimeras cannot produce offspring with *Prm1-* or *Prm2-*deficient alleles by natural mating, although sperm with *Prm1-* or *Prm2-*targeted alleles can be found in chimeras. Intracytoplasmic sperm injection (ICSI) also failed to obtain offspring using *Prm2-*deficient sperm. Previous researchers did not perform *in vitro* fertilisation using zone-free embryos. In both studies, they used the 129-derived ES cell line, whereas we used the TT2 ES cell line that were derived from BCF1 embryos. As previously reported^25^, the efficiency of ICSI in terms of the oocyte survival rate and the cleavage rate to the two-cell stage in the 129 strain were low. In addition, the phenotype analyses presented here suggest that the percentage of *Prm1*-deficient sperm that is compatible with development is low, which increases the difficulty of obtaining such sperm for ICSI.

In this study, we first used XO ES cells to produce female chimeras, which successfully produced male and female *Prm1*^+/−^ mice. Once we had obtained female *Prm1*^+/−^ mice, we could obtain male *Prm1*^+/−^ mice simply by mating the female *Prm1*^+/*−*^ mice with male *Prm1*^+/+^ mice. Alternatively, the sperm of *Prm1*^+/−^ mice could be used to obtain the next generation through IVF with zona-free oocytes. DNA damage induced in the sperm can be repaired in zygotes^26^,^27^,^28^, although matur sperm are incapable of repairing reactive oxygen species–induced DNA damage because these sperm lose their repair capabilities during spermatogenesis^29^. Generoso *et al.* demonstrated that paternal DNA can be repaired by maternal or zygotic factors^30^. Thus, the sperm DNA damage (if any) is not sufficiently severe to prevent the production of offspring and could be repaired after fertilization.

In sperm from the *Prm1*^+/−^ mice, the relative amount of PRM1 decreased, whereas the amount of mature PRM2 decreased and that of the precursor forms of PRM2 increased. The same results have been observed in sperm from chimeras^5^. These findings suggest that PRM1 is involved in the processing of PRM2. CMA3 and mBBr assays can be used to determine the protamine content of sperm chromatin. Interestingly, both the CMA3 and mBBr assays indicated biphasic peaks, suggesting the presence of two types of sperm; one with normal levels of PRM and another with lower levels. Transcription of *Prm1* and *Prm2* is initiated at step seven during spermiogenesis^31^, and these mRNAs are stored as cytoplasmic ribonucleoproteins before translation^32^. PRM1 synthesis starts in step 12 and PRM2 synthesis starts in step 13^2^. Although the genetically haploid spermatids are functionally diploid as a result of the sharing of gene products via their intercellular bridges^33^,^34^, PRM delivery from *Prm1*^+^ to *Prm1*^−^ sperm may not be sufficient because of late translation of PRM1 during spermiogenesis.

Like us, Cho *et al.*^5^ showed destabilisation of sperm DNA using the comet assay and SCSA. Both the comet assay and SCSA are thought to detect sperm DNA damage or integrity^22^,^35^, and DNA damage is considered to be the cause of infertility in *Prm1*^+/−^ mice^5^,^6^. However, we showed that viable offspring could be obtained from *Prm1-*deficient sperm using IVF and zona-free oocytes, suggesting that these methods can measure the stability and condensation of chromatin rather than critical DNA damage.

The infertility of *Prm1*^+/−^ mice might be caused not by critical sperm DNA damage but by reduced sperm motility resulting from an abnormal tail structure and disturbed energy metabolism. In fact, tail defects have frequently been reported as the cause of impaired motility and infertility. These defects include the absence of a definitive fibrous sheath and a shortened flagellum in mice deficient in the A-kinase anchoring protein 4^36^; flagella with abnormal axonemes and aberrant acrosomal structures in mice deficient in phospholipase A2, group III^37^; morphologically abnormal sperm with a frequent loss of the sperm head and disorganisation of flagellar structures, such as the loss of the central pair of microtubules and disorganization of the outer dense fibres and fibrous sheath, in mice deficient in sperm-associated antigen 6^38^; and defective microtubule structures of the axoneme in mice with a partial deletion of the chromosome 8 *β-defensin* cluster^39^. In addition, motility can be reduced by metabolic disturbance because functionally active mitochondria are essential for flagellar movement^40^. Thus, a reduction in MMP as revealed by JC1 staining may cause a decrease in sperm motility. Although protamine binding reportedly silences gene expression during spermiogenesis^41^,^42^,^43^, there was no obvious derangement of expression patterns in the absence of PRM1. Therefore, disturbed energy metabolism may not be caused by disordered global gene expression. Other proteins, such as PRM2 and transition protein, may protect these genes from aberrant expression. The relationship between *Prm1* deficiency and reduced sperm motility may be explained as follows. During spermatozoal maturation in the epididymis, the cysteines become progressively oxidized, thus forming inter- and intra-protamine disulfide bonds and further stabilizing both the chromatin^44^,^45^ and tail structures^46^,^47^. Zubkova *et al.*^48^ demonstrated that spermatozoa with fewer disulfide bonds caused by ageing are more susceptible to oxidative stress. Thus, *Prm1*-deficient spermatozoa may be more severely damaged by oxidative stress during maturation in the male reproductive tract. Taken together, these results suggest that exposure to oxidative radicals can cause changes in sperm morphology, thus leading to impaired spermatozoal motility.

AR was accelerated in the *Prm1*^+/−^ mice. In mammals, ejaculated sperm must complete capacitation to become competent to fertilise a mature oocyte. Capacitation involves several changes in the membrane properties and an increase in the intracellular calcium that drives motility and induction of the AR^49^. Only capacitated sperm can bind glycoproteins of the zona pellucida, undergo AR, and fertilise a mature oocyte. However, excessive intracellular calcium may induce microtubule defects and premature AR^39^. Such sperm will lose motility^50^ and the ability to firmly bind to the zona pellucida^39^ despite being capable of penetrating the egg^51^,^52^. It is currently unclear whether such an excessive increase in intracellular calcium level occurs in the absence of PRM1, and further studies will be required to address this question.

## Methods

### Generation of *Prm1* mutant mice

To construct the targeting vector, the *neo* cassette was inserted into the *Nae*I site of the first exon of *Prm1*. The lengths of the homologous regions in the *Prm1* targeting vector were 6.8 kb and 0.9 kb at the 5′ and 3′ ends, respectively, of the *neo* cassette. The 9-kb construct (5′-arm/*Neo*/3′-arm) for targeting was ligated into a pMCDT-A plasmid^53^ to generate the targeting vector. The targeting vectors were introduced into TT2-XO ES cells^20^ derived from an F1 embryo from a mating between C57BL/6 and CBA mice (Charles River Inc. Yokohama, Japan). G418-resistant clones were screened for homologous recombination by PCR, using the forward primer PAGN1 (5′-TCGTGCTTTACGGTATCGCCGCTCCCGATT-3′) in the *neo* gene and the reverse primer Prm-R2 (5′-ATATCTCTAGGTTTTCAGACGAGGCA-3′) on the 3′ end of the non-homologous region. The selected clones were confirmed by genomic Southern blot analyses (Supplementary Fig. 1). Targeted ES clones were injected into CD-1 eight-cell-stage embryos to generate chimeric mice. Foetuses and pups were genotyped by PCR using primers as described above. The mice were housed in an environmentally controlled room at the Center for Animal Resources and Development (CARD) at Kumamoto University. The experimental protocols that involved animals were approved by the Kumamoto University Ethics Committee for Animal Experiments (13–024) and all experiments were performed in accordance with the institute guidelines.

### *In vitro* fertilization after removal of the zona pellucida

Superovulation was induced in female wild-type CD-1 mice (Charles River. Yokohama.Japan) and oocytes were collected 14 to 16 h after human chorionic gonadotropin administration. The cumulus cells were removed from the oocytes by hyaluronidase treatment. To remove the zona pellucida, the oocytes were treated with acidic Tyrode’s solution^54^. Spermatozoa were isolated from the cauda epididymis and vas deferens of sexually mature mice and capacitated in HTF medium at 37 °C for 1.5 h. Spermatozoa (1 × 10^5^) were added to intact and zona-free oocytes and incubated for 6 h at 37 °C under 5% CO_2_. The eggs were cultured to the blastocyst stage. Blastocysts obtained after IVF were transferred into day-3 pseudopregnant females. Foetuses were collected from pregnant mice on day 12 after IVF.

### Sperm preparation

Spermatozoa obtained from age-matched adult mice were prepared from the cauda epididymis and vas deferens by gentle squeezing in mKSOM. Following the cell suspension was passed through a 70-μm mesh filter, it was centrifuged at 400 *g* for 5 min and washed twice with 2 mL of PBS. Spermatozoa were sonicated to detach the sperm heads from the tails if needed.

### Western blotting for sperm nuclear basic proteins

Sperm nuclear basic proteins were isolated and analysed as previously described^55^. A protease cocktail (P8340 Sigma) was used as the protease inhibitor. Gels were run and stained as previously described^56^. Proteins separated on the acid-urea gels were electroblotted onto an Immobilon-P^SQ^ filter (Millipore). Part of each blot was stained with 0.25% Coomassie brilliant blue. The other parts of the blots were incubated for 1 h at room temperature with anti-PRM1 antiserum or anti-PRM2 antiserum (kindly donated by Dr. Rodney Balhorn)^57^. The blots were washed three times for 15 min each and then incubated with anti-mouse IgG antibody linked to horseradish peroxidase. The bound complexes were *detected with ECL Plus reagents* (Amersham Pharmacia) and exposed to *x-ray film.*

### RT-PCR analysis

Total RNA was isolated from the cauda epididymis and vas deferens of the *Prm1*^+/+^ and *Prm1*^+/−^ mice using ISOGEN reagent (Wako, Tokyo, Japan). Total RNA was reverse transcribed using a SuperScript III First-Strand Synthesis System for RT-PCR (Invitrogen). Amplifications were performed for 24 cycles. The primers used were as follows: *Prm1* forward primer 5′-ATGGCCAGATACCGATGCTG-3′; *Prm1* reverse primer 5′-CTAGTATTTTTTACACCTTATGG-3′; *Prm2* forward primer 5′-ATGGTTCGCTACCGAATGAGG-3′; *Prm2* reverse primer 5′-TTAGTGATGGTGCCTCCTACA-3′; *Tnp1* forward primer 5′-ATGTCGACCAGCCGCAAGC-3′; *Tnp1* reverse primer 5′-TCACAAGTGGGATCGGTAATTG-3′; GAPDH (housekeeping gene) forward primer 5′-TGTCATCAACGGGAAGCCCA-3′; and GAPDH reverse primer 5′-TTGTCATGGATGACCTTGGC-3′.

### CMA3 staining

CMA3^58^ staining was performed as previously described^59^ with some modifications. Briefly, spermatozoa were incubated in staining solution for 20 min at room temperature in the dark.

### mBBr thiol labelling assay

Thiol labelling was performed using mBBr as previously described^48^. Briefly, sperm were resuspended in PBS and aliquots were incubated with or without 1 mM DTT to reduce sperm disulphides to reactive thiols. The samples were then washed twice and resuspended in PBS. A solution of mBBr was added to the sperm suspension for a final mBBr concentration of 1 mM, and the sample was incubated in the dark. The sperm were then washed in PBS and sonicated on ice in the dark until analysis.

### Comet assay

A comet assay was performed by using a CometAssay kit (Trevigen). Briefly, spermatozoa (1 × 10^5^ cells/mL) in PBS were mixed with melted agarose and then immediately pipetted on to a CometSlide. The slides were immersed for 1 h on ice in pre-chilled lysis buffer, incubated for 18 h in lysis buffer containing 500 μg/mL proteinase K^60^. and then subjected to electrophoresis. Finally, the slides were immersed in chilled 70% ethanol and was stained with SYBR Green. The results were expressed as the ‘tail length’ and ‘% DNA in tail’, using TriTek Comet Score-Freeware v1.5 (TriTek Corp., Sumerduck, VA, USA).

### Sperm chromatin structure assay (SCSA)

Washed fresh sperm samples were diluted with TNE buffer to obtain a concentration of 1 to 2 × 10^6^ cells/mL. A 0.2-mL aliquot was then mixed with 0.4 mL of acid detergent. Thirty seconds later, 1.2 mL of AO staining solution was added^22^,^61^. The fluorescence of individual nuclei was measured with a FACSAria cytometer (BD Biosciences).

### Fluorescence and flow cytometry analysis

Fluorescently labelled spermatozoa were analysed using fluorescence microscopy and a FACSAria flow cytometer (BD Biosciences). Data were processed with BD FACSDiva and FlowJo software. Data are representative of three or more experiments with over 5000 cells.

### Sperm morphology

Sperm samples were fixed in buffered formol–saline and stained with eosin according to standard procedures.

For the SEM analysis, the specimens were fixed for 1 h with 2% glutaraldehyde in 0.1 M phosphate buffer (pH 7.4). A drop of sperm suspension was then adhered to a poly-L-lysine-coated cover slip, which was washed three times with PBS. The specimens were dehydrated in a graded ethanol series and then freeze dried in a *t*-butanol freeze-drying apparatus (JEOL, JFD-320). The dried specimens were sputter-coated with platinum by an ion coater (JEOL, JFC-1600) and observed by SEM (Keyence, VE-9800).

For the TEM analysis, the specimens were fixed with 2% glutaraldehyde, post-fixed in 1% OsO_4_, dehydrated through a graded ethanol series, and embedded in Quetol 812 (Nissin EM Co.). Ultra-thin sections were counterstained with uranyl acetate and lead citrate and then observed under a TEM (model H7100, Hitachi, Tokyo, Japan) at a 75-kV accelerating voltage^62^.

### Sperm motility

Sperm motility was evaluated immediately by placing a 10-μL drop of diluted sperm suspension between a glass slide and a cover slip and counting the sperm under a phase-contrast microscope at ×1000 magnification. Sperm motility was assessed as the % motile cells.

### Assessment of acrosomal reaction

The AR was assessed according to a previously described method^63^. Mouse sperm was incubated in a 37 °C water bath to allow capacitation. Then, the Ca^2+^ ionophore A23187 was added to induce the acrosomal reaction. Wet-mount preparations of semen samples were fixed in buffered formol–saline and the acrosome region was stained with 0.22% Coomassie brilliant blue before and after incubation at 37 °C for 90 min. Acrosomal staining was scored under a bright-field microscope. Ninety cells were counted in three wild-type and three *Prm1*^+/−^ mice.

### JC-1 staining

JC-1 dye can be used as an indicator of MMP in a variety of cell types, including spermatozoa. JC-1 dye exhibits a potential-dependent accumulation in mitochondria, as indicated by a red fluorescence emission (~590 nm). Thus, mitochondrial depolarization is indicated by a decrease in the red/green fluorescence intensity ratio. JC-1 staining was performed as previously described^59^.

### DNA microarray analysis

RNA was isolated from sperm collected from the cauda epididymis and purified using an RNeasy Lipid Tissue Mini Kit (Qiagen, Valencia, CA). Total RNA (100 ng) was used to synthesise double-stranded cDNA (dsDNA). Anti-sense cRNA was synthesized from the dsDNA template and subsequently used to produce sense single-stranded cDNA (ssDNA). The ssDNAs were fragmented, end-labelled, and hybridised to a Genechip Mouse Transcript Assay 1.0 set (Affymetrix, Tokyo, Japan). Arrays were hybridised and scanned using a standard protocol. Microarray data were analysed using an Affymetrix Expression Console and Affymetrix Transcriptome Analysis Console (Affymetrix, Tokyo, Japan).

### Number of mice used for each experiment

The number of mice used for each experiment is shown in Supplementary Table 2.

### Statistical analysis

The comet parameters were analysed using the Mann-Whitney U-Test. In other experiments, statistical analyses were performed with unpaired Student’s *t*-tests. The results are expressed as the means ± SE. Differences were considered statistically significant at *P* < 0.05.

## Additional Information

**How to cite this article**: Takeda, N. *et al.* Viable offspring obtained from *Prm1*-deficient sperm in mice. *Sci. Rep.* **6**, 27409; doi: 10.1038/srep27409 (2016).

## Supplementary Material

Supplementary Information
