# Supplementary material for: Viable offspring obtained from Prm1-deficient sperm in mice
Source: Sci Rep. 2016 Jun 2;6:27409. doi: 10.1038/srep27409 (PMC4890041; doi:10.1038/srep27409)
Supplement: Supplementary Information [file srep27409-s1.pdf]

Supplementary Information for

Viable offspring obtained from *Prm1*-deficient sperm in mice

Naoki Takeda[1], Kazuya Yoshinaga, Kenryo Furushima, Kazufumi Takamune, Zhenghua Li, Shin-ichi Abe, Shin-ichi Aizawa and Ken-ichi Yamamura*

Contents

Supplementary Figure 1
Supplementary Table 1
Supplementary Table 2

Supplementary Figure 1

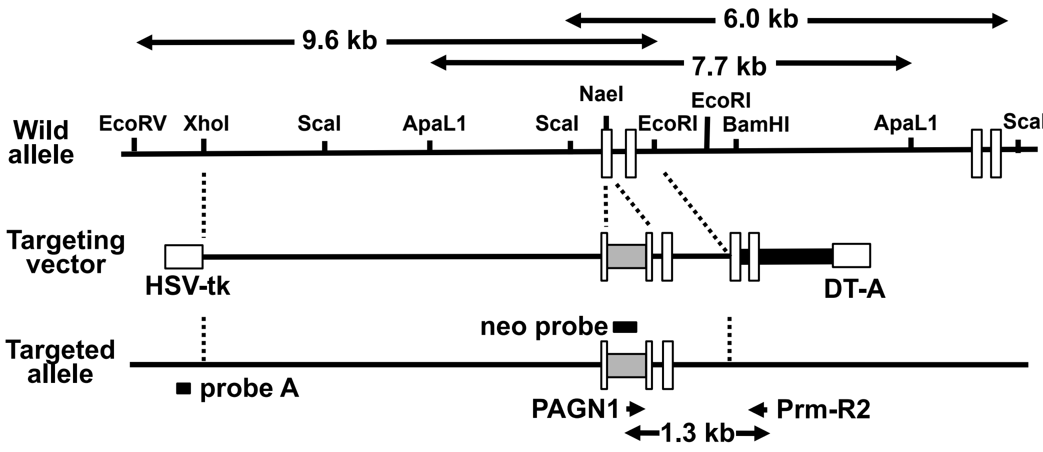

Supplementary Figure 1. Targeted mutation at the *Prm1* locus. The positive neomycin selection cassette (*neo*) was inserted into the *Nae*I site within the first exon. The diphtheria toxin A fragment (DT-A) gene was used for negative selection. The locations of probe A and the *neo* probe used for the Southern blot analyses are shown, and the expected sizes of the hybridizing fragments are presented above.

Supplementary Table 1

Supplementary Table 1. Macroscopic findings in the *Prm*$^{+/–}$ *and Prm*$^{+/–}$ male mice.

	Prm$^{+/+}$ (n = 6)	*Prm*$^{+/–}$ (n = 7)
Body weight (g)	23.5 ± 0.6	23.8 ± 0.4
Testis (mg)	72.7 ± 1.1	78.5 ± 1.2
Epididymis (mg)	27.4 ± 0.5	30.9 ± 0.7

Data are presented as the mean ± s.d.

Supplementary Table 2

Supplementary Table 2. Number of mice used for each experiment.

Figure	Experiment	Age	*Prm+/+*	*Prm+/-*	cell number
Fig. 1a	RT-PCR	4 months	3	3	-
Fig. 1b	Western blot	5 months	2	2	-
Fig. 2a	CMA3 staining	8 months	2	2	over 5000
Fig. 2b & c	mBBr thiol labelling assay	9 months	3	3	over 5000
Fig. 3a & b	Comet assay	7 months	3	3	wild = 12, hetero = 19
Fig. 3c & d	Sperm chromatin structure assay	8 months	3	3	over 5000
Fig. 4a to h	Eosin staining	11 months	2	2	-
Fig. 4i to n	Scaning electron microscopy	11 months	3	3	-
Fig. 4o	Morphology	12 months	3	3	wild = 573, hetero = 453
Fig. 5	Transmission electron microscopy	3 months	2	2	over 5000
Fig. 6a	Acrosomal reaction	12 months	3	3	0 min (wild = 327, hetero = 365), 90 min (wild = 573, hetero = 453)
Fig. 6b	Motility	9 to 12 months	6	7	wild = 1204, hetero = 1711
Fig. 6c & d	JC-1 staining	6 months	4	3	over 5000
Fig. 6e	DNA microarray analysis	5 months	3	3	-
